# Association of Leptin and Leptin receptor Gene polymorphisms with Insulin resistance in pregnant women: A cross-sectional study

**DOI:** 10.12688/f1000research.122537.1

**Published:** 2022-06-22

**Authors:** Usha Adiga, Nandit Banawalikar, Tirthal Rai

**Affiliations:** 1 Department of Biochemistry, KS Hegde Medical Academy, Nitte-DU, Mangalore, 575018, India; 2 Rivaara Lab Pvt Ltd, Bangalore, India

**Keywords:** leptin, Leptin receptor, single nucleotide polymorphism, insulin resistance, pregnancy

## Abstract

**Introduction:** Leptin, along with its receptor, are linked with mechanisms affecting a diverse array of pregnancy-specific pathologies that include gestational diabetes and intrauterine growth restriction. The goal of the study was to examine if there was a link between the leptin (LEP)/leptin receptor (LEPR) gene polymorphism and insulin resistance in pregnant women, and to determine the extent to which the leptin gene polymorphism could cause insulin resistance..

**Methods:** 208 pregnant women participated in this cross-sectional study of which 74 were insulin resistant cases and 134 were insulin sensitive controls. The study was carried out from December 2018 to December 2020 at a charitable hospital in Mangalore, Karnataka, India. Genotyping of leptin and its receptor gene were carried out using the Polymerase Chain Reaction- Restriction fragment Length Polymorphism (PCR-RFLP) method. Serum levels of leptin, insulin, and C peptide were assayed using Enzyme Linked Immuno Sorbent Assay (ELISA). Statistical analysis was carried out using SPSS 23.

**Results:** Insignificant association was observed between leptin receptor gene polymorphisms and insulin resistance, and leptin gene and insulin resistant women. There was no significant difference in the serum leptin levels among the cases and control (61.62±29.23 and 59.88±22.25). However, fasting blood sugar, insulin, C peptide, Triglycerides (TG), and very low-density Lipoprotein (VLDL) levels were significantly higher in cases as compared to controls (p=0.0068, p<0.0001, p<0.0001 and 0.01 respectively). Homeostatic Model Assessment for Insulin Resistance (HOMA IR) was greater in subjects with homozygous dominant, 'GG' of LEPR (p=0.0409) and hyperinsulinemia (p=0.023) as compared to other genotypes. However, hyperglycaemia was observed in subjects with homozygous dominant, ‘AA’ of leptin gene (p=0.0173).

**Conclusion:** No significant association was found between leptin and leptin receptor gene polymorphisms with insulin resistance in pregnancy. However, genotyping of these genes may be useful in predicting insulin resistance and gestational diabetes in pregnancy.

## Introduction

Pregnancy is often accompanied by growing insulin resistance that begins in the second trimester and escalates to levels comparable to type 2 diabetes insulin resistance in the third trimester. Insulin resistance and hyperinsulinemia may be the common ground for the pregnancy metabolic syndrome.

Insulin is a biologically significant peptide produced mostly by the pancreas' beta cells. Many somatic cells have membrane receptors that bind to this hormone and coordinate its involvement in nutrient availability. Insulin plays a concentration-dependent function in glucose elimination, as evidenced by its direct action on tissues such as the liver, skeletal muscle, and adipocytes, all of which contribute to metabolic balance. ^1^ Insulin responsiveness refers to the insulin concentration required for maximum response, while insulin sensitivity refers to the half-maximal response. ^2^ Insulin resistance, on the other hand, occurs when cells partially or totally stop reacting to insulin. This frequently results in long-term hyperglycaemia, type 2 diabetes, hyperinsulinemia, dyslipidaemia, hypertension, and atherosclerosis. ^3^ The clinically useful surrogate markers of insulin resistance are Homeostatic Model Assessment for Insulin Resistance (HOMA-IR), Homeostatic Model Assessment 2 (HOMA2), Quantitative Insulin Sensitivity Check Index (QUICKI), serum triglyceride, and triglyceride/High Density Lipoprotein (HDL) ratio.

Leptin (LEP) is a 167-amino-acid adipokine produced by the Lep gene, which is found on chromosome 7 (7q32.1). It is mostly released by adipocytes, which explains why it correlates with body mass, and it is minimally secreted by the placenta, stomach, and intestine. It regulates hunger, boosts energy expenditure, and affects insulin production, insulin sensitivity, and glucose homeostasis, among other things. ^4^

Insulin and leptin are interrelated regulatory hormones in pregnancy; leptin modulates insulin sensitivity exclusively by modulating gluconeogenesis in the liver and insulin-dependent glucose metabolism in the skeletal muscles. ^5^ In human trophoblastic cells, insulin stimulates foetal growth in the uterus by establishing a glucose-induced gradient between maternal and foetal insulin production, whereas leptin stimulates cell proliferation, protein synthesis, and apoptosis inhibition. Leptin binds to the leptin receptors (LEPR) (1165-amino-acids), the gene for which is located on chromosome 1p31. The 2548th position of the LEP gene polymorphism is produced by the substitution of glutamine for arginine, while the 223rd position of the LEPR gene polymorphism is caused by the substitution of glutamine for arginine.

Both insulin and leptin receptors share a common signal transduction pathway, the modifications of any of these have the potential to change the metabolic condition of the mother and contribute to foetal obesity. According to the literature, increased leptin levels influence the activation of insulin signalling, which affects insulin sensitivity in later stages of pregnancy. ^6^^,^^7^ This decreased insulin sensitivity can progress to gestational diabetes mellitus (GDM). Studying the leptin/LEPR gene polymorphism in insulin resistant pregnant women versus insulin sensitive pregnant women is justified. Leptin - leptin receptor - inslin resistance - lipid profile axis which may have a substantial role in the pathogenesis of GDM is least explored in the Indian population.

### Objectives

The goal of the study was to examine if there was a link between the leptin (LEP) (rs77990) as well as LEPR gene (rs1137101) with insulin resistance and lipid profile, in order to determine the extent to which the leptin gene polymorphism could cause insulin resistance in pregnant women. The study also aimed to compare the serum insulin, leptin, lipid profile, and C peptide levels between insulin resistant and insulin sensitive pregnant women.

## Methods

### Ethical statement

Approval for this study was obtained from the Central Ethics Committee, Nitte Deemed to be University (NU) of which KS Hegde Medical Academy is a constituent College (NU/CEC/2018/01). Eligible patients were given information on the study's goals, procedures, and role in the study, as well as a consent form (in their native language). Following their agreement, written informed consent was obtained.

### Study design and setting

In December 2018-December 2020, a cross-sectional study was undertaken on pregnant women who visited the Department of Obstetrics and Gynecology at a charitable hospital in Mangalore, Karnataka, India. The blood samples were examined at the K.S.Hegde Medical Academy's Central Research Laboratory's Endocrinology and Molecular Genetics wing.

### Study subjects

The study enrolled 208 pregnant women who visited the Department of Obstetrics and Gynecology at a charitable hospital in Mangalore, Karnataka, India agreed to take part after obtaining their informed consent. The homeostasis model assessment model (HOMA) was used to determine insulin resistance. The pregnant women were divided into two groups, insulin resistant (group 1) and insulin sensitive (group 2), with 74 being insulin resistant and 134 being insulin sensitive (HOMA-IR < 2.5 is considered as insulin sensitive as per the standard published literatures). Understanding the contribution of insulin resistance to the pathogenesis of gestational diabetes is important for establishing preventive measures and determining optimal therapeutic approaches. As the grouping was done after recruiting patients, the number of subjects was asymmetric in two groups. However, selection of the subjects was not biased and the age and BMI (pre-pregnancy) of the subjects in both the groups were matched. The gestational age of subjects were also not significantly different (25.3 ± 1.11 weeks and 25.8 ± 1.34 weeks in insulin resistant and insulin sensitive subjects respectively).

Exclusion criteria: Multiple pregnancies, pre-gestational diabetes, pregnancy-induced hypertension, foetal abnormalities, and known heart, renal, or liver diseases were all ruled out based on the medical history, clinical examination, and basic laboratory investigations. In addition to this, a proforma was utilised to obtain general demographic information, family history of hypertension, and diabetes.

### Data collection and analysis of blood samples

Five millilitres (ml) of blood was drawn from each participant, after eight hours of fasting. Two ml were used for biochemical analysis and three ml for genetic study. Fasting samples were taken in plain vacutainers for leptin, insulin, C-peptide, and lipid profile analyses, as well as a plasma sample for fasting blood sugar analysis. The ELISA method was used to measure leptin, insulin, and C-peptide using reagent kits manufactured by Immunoconcept India Pvt Ltd with a sensitivity of 98% and specificity of 96%. Intra and inter assay coefficients of variation of leptin and C-peptide ranged from 1.8-2.4%. The lipid profile and fasting blood sugar levels, on the other hand, were examined in the Cobas C311, a fully automated chemistry analyzer. The conventional HOMA-IR formula was used to calculate insulin resistance:

$$\text{HOMA}-\mathrm{IR}=\left(\text{fasting glucose}\;\times \;\text{fasting insulin}\right)/22.5;\text{insulin expressed in}\;\mu \;U/L,\text{glucose in mmol}/l.$$

### Genetic analysis

For DNA extraction from leucocytes, a DNA extraction mini kit (Thermofishers scientific, catalogue number: K182001) was employed, and the purity of the extracted DNA was verified using electrophoresis. For electrophoresis, a 0.8%Agarose gel with 0.5 g/ml ethidium bromide in TAE buffer was utilised. The purity of the DNA was determined using a spectrophotometer (OD260/OD280 ratio).

Leptin, LEP G2548A alleles (rs77990), and leptin receptor, LEPR Gln223Arg alleles (rs1137101) were genotyped using Polymerase Chain Reaction-Restriction Fragment Length Polymorphism (PCR-RFLP) using appropriate forward and reverse primers. The resulting product was digested using appropriate restriction enzymes. Ethidium bromide staining was used to visualize the reaction mixtures after electrophoresis on a 2% agarose gel. Table 1 shows the primers and restriction enzymes that were employed.

**Table 1.** Details of Polymerase Chain Reaction- Restriction Fragment Length Polymorphism (PCR-RFLP) for the gene Leptin and Leptin Receptor (LEPR).

SNP	Location (Base change)	Forward primer Reverse primer	PCR program (35 cycles)	PCR fragment length (Bp)	Restriction enzyme, Incubation temperature	Allele: RFLP fragment size
LEP (rs7799039)	Promoter (G>A)	5’-TTTCCTGTAATTTTCCCGTGAG-3’ 5’AAAGCAAAGACAGGCATAAAAA-3’	93°C,45’, 61°C,30’, 72°C,30’	242	HhaI, 37°C	Allele A:242 Allele G:181+61
LEPR (rs1137101)	Exon 6(A>G)	5’-AAACTCAACGACACTCTCCTT-3’ 5’-TGAACTGACATTAGAGGTGAC-3’	93°C,45’, 57°C,30’, 72°C,30’	80	MspI, 37 °C	Allele A:80 Allele G:59+21

### Statistical analysis

SPSS 23.0 ( https://www.ibm.com/support/pages/downloading-ibm-spss-statistics-23) was used to conduct the statistical analysis. Continuous data was expressed as mean standard deviation, whereas categorical data was expressed as percentage and frequencies. Hardy-Weinberg Equilibrium (HWE) was used to examine the distribution of allele frequencies between different variations for the LEP and LEPR gene variants among cases, and the chi-square test was used to evaluate the distribution of allele frequencies between different variants. The link between leptin gene polymorphism distribution and insulin resistance was assessed using the Chi-square test. The biochemical characteristics of insulin resistant individuals and insulin sensitive controls were compared using an unpaired t test. The association between biochemical markers and insulin resistance was determined using Pearson's correlation test. The chance of a leptin gene variation causing insulin resistance was calculated using Odd's ratio. Comparison of biochemical parameters between the alleles was done by One Way ANOVA. ‘p’ value <0.05 was regarded as statistically significant.

## Results

A total of 208 pregnant women were included in this study. Insulin resistant women of 28.91 ± 4.125 years in group 1 and insulin sensitive women of 27.93 ± 4.23 years in group 2 were recruited in the study.

### Leptin gene and receptor polymorphism pattern

**Figure 1.**
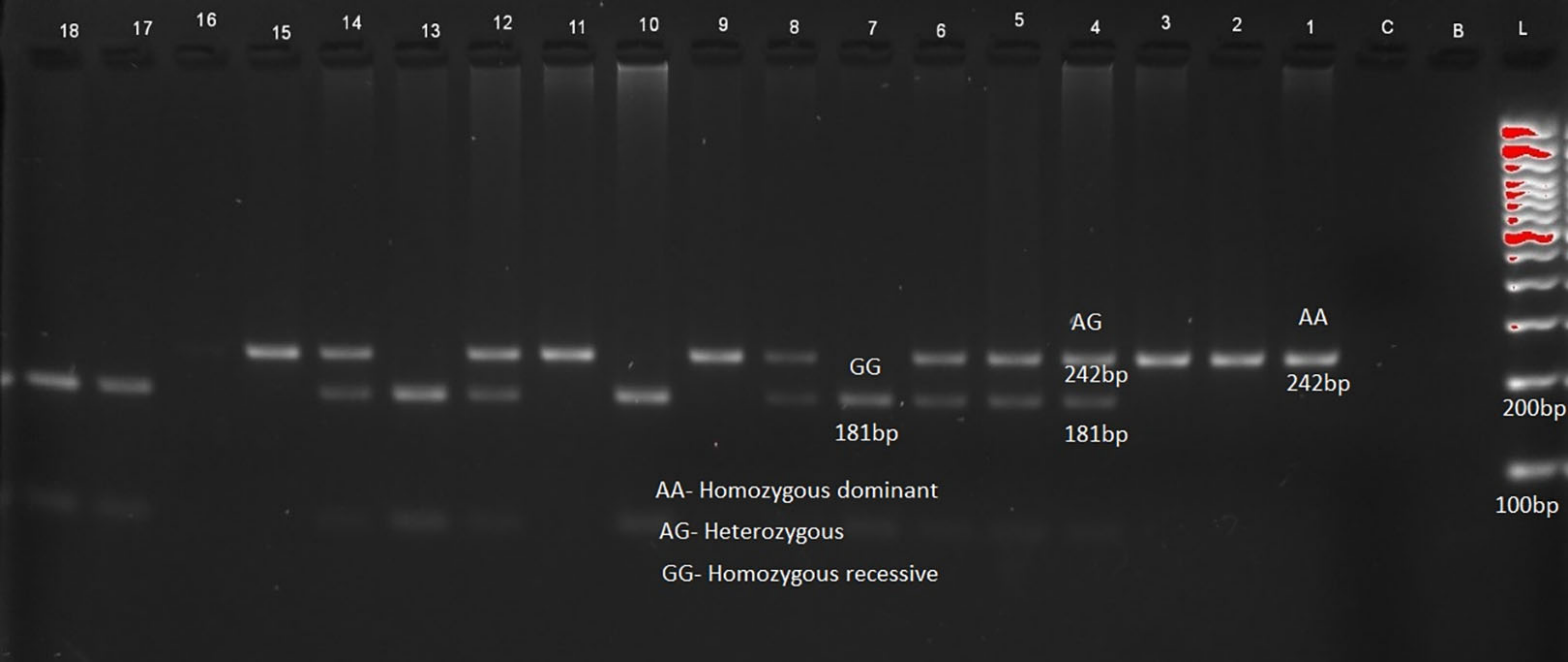
Sample pattern of distribution of alleles of leptin gene (LEP).

**Figure 2.**
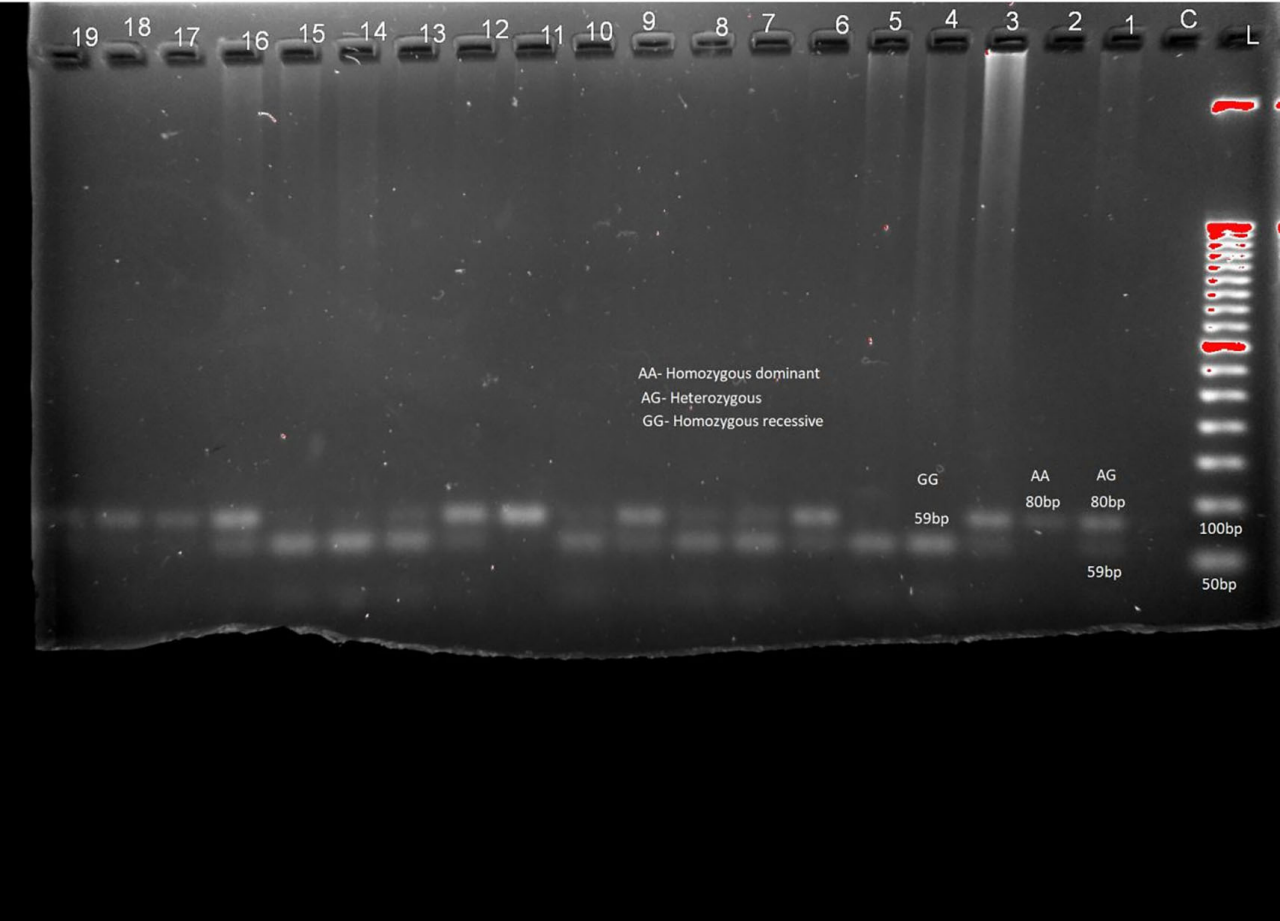
Sample pattern of distribution of alleles of Leptin receptor gene (LEPR). Association studies among insulin resistance and genes for leptin were carried out. No significant association was found between them (chi-square 0.221, p = 0.638 ( Table 3). There was also a non-significant link between Leptin receptor gene polymorphisms and insulin resistance (chi-square value = 0.1275, p value = 0.721). In patients with alleles for the leptin/LEPR genes, odd's ratio revealed no risk of insulin resistance ( Table 3).

The distribution of genotypes and alleles of LEP (rs7799039) in Table 2 and Figure 1, LEPR (rs1137101) gene variants are provided in Table 2 and Figure 2 respectively. RFLP patterns were interpreted as homozygous dominant (AA) (wild type) **,** heterozygous (AG), homozygous Recessive (GG), both being mutant alleles, based on the location and intensity of the bands ( Figure 1 and Figure 2), frequencies of which are represented in Table 2. Frequencies of both wild and mutant type of alleles was found to be higher among insulin sensitive subjects. None of the genotype frequency distributions for rs7799039 variants deviated significantly from HWE in insulin resistant cases (p > 0.05). However, the frequency for allelic distribution for rs1137101 variants showed significant deviation of observed from expected in insulin resistant cases, suggesting that alleles were not in equilibrium.

**Table 2.** Hardy-Weinberg Equilibrium (HWE) for the LEP gene and LEP receptor.

Frequency of pattern in LEP gene (rs7799039)			Chi -square value for LEP	Frequency of pattern in LEPR gene (rs1137101)		Chi -square value for LEPR
IR (Gp1)		Insulin sensitive (Gp2)		IR (Gp1)	Insulin sensitive (Gp2)	
AA *	21	34	0.473 (Gp1) p- >0.05 0.507 (Gp2) p- >0.05	33	39	7.558 (Gp1) P<0.05 0.568 (Gp2) p>0.05
AG *	36	69		24	65	
GG *	17	31		17	30	

**Notes:** LEP Gene -Cases: Frequency range – ‘A’ allele -0.545; ‘G’ allele – 0.45; Controls: p allele frequency: 0.507, q allele frequency: 0.4928. LEP Receptor: Cases: Frequency range – ‘A’ allele -0.48; ‘G’ allele – 0.515. Controls: p allele frequency: 0.4856, q allele frequency: 0.5144 Statistical method used: HWE,Chi square.

* AA: Homozygous dominant, AG: Heterozygous, GG: Homozygous Recessive.

Fasting blood sugar (p = 0.0068), insulin, triglycerides, VLDL and C peptide levels in group 1 were significantly higher in comparison to subjects in group 2 (p < 0.0001). Fasting leptin levels were insignificantly higher in insulin resistant cases as compared to the sensitive (61.62 ± 29.23 and 59.88 ± 22.25). Lipid profile indicators such as total cholesterol, low density lipoprotein, and high-density lipoprotein levels did not differ significantly between the groups as shown in Table 4. Serum leptin showed highly significant negative correlation with Homeostatic Model Assessment for Insulin Resistance (HOMAIR) (r = -0.316) (p-0.006), whereas C peptide showed high significant negative correlation with total cholesterol (r = -0.394), LDL(r = -0.372) and positive significant correlation with HOMAIR (r = 0.283) ( Table 5) with p values being 0.001, 0.001, and 0.015 respectively ( Table 4).

**Table 3.** Association between leptin gene and leptin receptor gene polymorphism in insulin resistant and insulin sensitive pregnant women.

Gene variant	LEP gene (rs7799039)			Chi-square value *	LEPR gene (rs1137101)			Chi-square value
	Insulin resistant	Insulin sensitive	Total		Insulin resistant	Insulin sensitive	Total	
GG	17	36	53	0.470 p = 0.638	17	35	52	0.357 p = 0.721
AG + AA	57	103	160	Odds Ratio = 0.853	57	104	161	Odds ratio = 0.886
	74	139	213		74	139	213	

* Statistical method used: Chi square.

**Table 4.** Demographic and biochemical parameters in insulin resistant and insulin sensitive women.

Parameter	Insulin resistant cases	Insulin sensitive Controls	p value
Mean age (years)	28.91 ± 4.125	27.93 ± 4.23	>0.05
BMI (kg/m ^2^)	25.86 ± 5.86	25.78 ± 6.84	>0.05
FBS sugar (mg/dl)	120.7 ± 34.99	90.58 ± 24.4	0.0068**
Fasting Insulin μIU/L	17.73 ± 11.76	2.19 ± 1.86	<0.0001**
C-peptide (nmol/L)	2.51 ± 1.90	1.57 ± 1.55	<0.0001**
Leptin (ng/ml)	61.62 ± 29.23	59.88 ± 22.25	0.632
TG (mg/dl)	263 ± 105.3	231.6 ± 70.87	0.010 *
TC (mg/dl)	225.2 ± 38.83	228.2 ± 52.37	0.67
HDL (mg/dl)	53.58 ± 11.72	52.83 ± 12.63	0.671
LDL (mg/dl)	147.7 ± 42.58	154.4 ± 49.9	0.325
VLDL (mg/dl)	51.65 ± 20.19	46.32 ± 13.68	0.026 *
HOMAIR	5.45 ± 4.6	0.549 ± 0.52	<0.0001**

Statistical method used: Unpaired t test.

* p < 0.05 significant.

**Table 5.** Correlation of serum leptin, C peptide, and HOMAIR with various parameters in all subjects.

	HOMAIR	C Peptide	Leptin	TG	Cholesterol	HDL	LDL	VLDL
	Pearson’s coefficient (r)							
**C Peptide**	0.283 *	1	-0.223	0.119	-0.394**	-0,209	-0.372**	0.122
**Serum leptin**	-0.316**	-0.223	1	0.111	0.092	0.020	0.024	0.080
**HOMAIR**	1	0.283 *	-0.316**	0.163	-0.067	-0.173	-0.107	0.180

Statistical method used: Pearson’s correlation.

Abbreviations: TG, Triglycerides; HDL, high density lipoprotein; LDL, low density lipoprotein; VLDL, very low-density lipoprotein.

* p < 0.05 significant.

On comparing biochemical markers among subjects of group 1, insulin, C peptide, leptin, and lipid parameters did not differ significantly between the genotypes AA, GG, and AG of the leptin gene and a significant difference in fasting blood sugar was observed (p = 0.0173) ( Table 6). C peptide, leptin, and lipid indicators showed no significant differences when compared with different genotypes of the leptin receptor (Lep R) gene, homozygous recessive AA, heterozygous AG, and homozygous dominant GG. However, a significant difference in insulin levels was observed (p value-0.023) ( Table 6).

**Table 6.** Comparison of parameters between three alleles of leptin receptor gene.

	Homozygous recessive AA	Heterozygous AG	Heterozygous dominant GG	P value
**FBS (mg/dl)**	109.4 ± 33.07	113.6 ± 35.6	111.9 ± 34.98	0.0173
**INSULIN (μIU/L)**	8.37 ± 13.22	5.495 ± 6.7	9.97 ± 11.13	0.023 *
**C PEPTIDE (nmol/L)**	1.65 ± 1.21	2.04 ± 1.86	1.79 ± 1.65	0.374
**LEPTIN (ng/ml)**	62.92 ± 24.8	58.95 ± 24	60.60 ± 26.25	0.664
**TRIGLYCERIDES (mg/dl)**	234.1 ± 89.7	252 ± 92.79	236.8 ± 71.99	0.385
**CHOLESTROL (mg/dl)**	229.6 ± 48.22	228.8 ± 49.8	223.4 ± 46.06	0.719
**HDL (mg/dl)**	52.27 ± 13.38	52.79 ± 11.52	54.06 ± 12.53	0.696
**LDL (mg/dl)**	155.7 ± 47.22	151.2 ± 49.61	150.7 ± 45.52	0.824
**HOMAIR**	2.47 ± 4.3	1.56 ± 2.25	2.96 ± 4.21	0.0409 *

Statistical method used: One way ANOVA.

* p < 0.05 significant.

## Discussion

The goal of this study was to determine the pattern of leptin gene and receptor polymorphism in insulin resistant and insulin sensitive pregnant women, as well as its relationship to insulin resistance. Biochemical indices like fasting insulin, C peptide, sugar and lipid parameters and their correlation were analysed.

For the LEP G2548A polymorphism, the frequency of A alleles was greater in insulin resistant pregnant women, while the frequency of G alleles was higher in insulin sensitive pregnant women in our study. In insulin resistant pregnant women, the frequency of A allele was likewise insignificantly higher in the LEPR Gln223Arg polymorphism. Similar studies on GDM patients, however, have revealed a similar trend of 'A' allele frequency preponderance. ^8^ A study found contradictory results, with a greater G allele frequency. ^9^ In another, GDM risk was increased in patients with the AA and AG genotypes, according to Vasku *et al.* ^4^

The results in our study reported no significant association between LEP G2548A and LEPR Gln223Arg polymorphism in insulin resistant and insulin sensitive pregnant women ( Table 3). Unfortunately, there is no concrete evidence to support these results as there a no putative studies done on insulin sensitive and resistant pregnant women, although Ying *et al.,* found association between LEPR Gln223Arg gene polymorphism and T2DM with higher ‘A’ allele frequency in them. ^10^ Another recent study demonstrated higher ‘AA’ genotype of LEPR in insulin resistance patients. ^11^ By contrast, Fang *et al.,* Shi *et al.,* and a meta-analysis done by Li *et al.,* claimed a greater risk for T2DM in subjects with G allele of LEPR Gln223Arg gene polymorphism. ^12^^–^^14^ Even though the frequency of A allele of LEPR Gln223Arg polymorphism was higher in insulin resistant women in our study group, these findings did not suggest any risk of developing insulin resistance in pregnancy ( Table 3).

There was no significant change in serum leptin levels between insulin resistant and insulin sensitive pregnant women. However, utilising a conventional reference range of 2.5-21.8 ng/ml for serum leptin as a guideline, hyperleptinemia was seen in both the groups. Due to increased fat content and the influence of the placenta, which accelerates maternal leptin levels at 28 weeks of gestation, all mammalian pregnancies have a 2-3-fold increase in serum leptin levels. ^15^ When leptin levels were evaluated among insulin resistant individuals with different genotypes, patients with the 'AA' allele had minor hyperleptinemia. Ren *et al.,* on the other hand, discovered that in type 2 diabetes patients with the AA genotype, plasma levels of leptin and insulin were much lower than those with the GA or GG genotypes. ^16^

The G2548A polymorphism in leptin (LEP) has been linked to enhanced leptin synthesis and plasma release by adipocytes. Leptin functions through receptors, and a Gln223Arg mutation in leptin receptors (LEPR) impairs signalling capacity. ^5^^,^^6^

Hyperleptinemia was observed in insulin resistant subjects (though insignificant). Fasting insulin and C peptide were higher in insulin resistant cases in the study. Leptin inhibits insulin secretion by causing the release of leptin-induced proinflammatory cytokines such as C-reactive protein and Interleukin-6, which promote pancreatic-cell death. ^17^ Leptin levels confirm a negative link with insulin levels, and our investigation does not support this idea. This contradictory finding could be due to leptin resistance.

In our study, however, insulin resistant women had significantly higher serum insulin and C peptide levels than insulin sensitive women. This is owing to the different roles of insulin in different people, since it induces glucose to influx into tissues, and these tissues have a decreased responsiveness to insulin activity in insulin resistance. As a result, in insulin resistant people, the pancreas produces too much insulin to compensate for their resistance (2). C peptide is a well-known biomarker of insulin production because it is likewise secreted by the pancreas' beta cells and is used to diagnose insulin resistance because it has a significantly longer half-life than insulin. Hyperinsulinemia and increased C peptide levels are thus linked in insulin resistance. ^18^

In our investigation, leptin had a strong and significant negative association with HOMAIR, but C peptide had a strong and significant positive correlation with HOMAIR, which is supported by Khan HA *et al.* ^18^ In contrast to our findings, investigations in pregnant women showed a favourable connection between leptin and the HOMA index. ^15^ While Das *et al.,* found a link between leptin and HOMA-IR levels in T2DM patients, ^19^ another investigation found a substantial link between insulin resistance and morbid obesity in morbidly obese women. ^20^ As both leptin resistance and IR are dependent on adipose metabolism, obesity is a major pathogenic factor in both. ^21^

As there is substantial weight gain and increased leptin release through the placenta in pregnancy, regardless of insulin resistance, leptin levels in pregnancy are debatable. However, no significant differences in C-peptide and insulin levels were found in our investigation among three alleles of the leptin gene. Nonetheless, patients with the ‘GG’ allele of the leptin receptor gene had significantly higher serum insulin and HOMAIR, according to our research.

Although it is well known that insulin resistance leads to hyperglycaemia, leptin also plays a role in glucose homeostasis by acting directly on pro-opiomelanocortin neurons. ^22^ Because leptin receptors are found in the pancreas, leptin suppresses insulin release from beta cells, whereas insulin increases leptin production from adipose tissue.

The dysfunction of this hormonal regulatory feedback loop was found to increase insulin resistance and hyperglycaemia. ^23^ The fasting blood glucose level in the present study was significantly higher in insulin resistant pregnant women as compared to the controls. Our findings also observed that among the three LEP G2548A polymorphism alleles ‘AA’ showed significantly higher glucose level as compared to other genotypes.

In our study, there was a significant increase in triglycerides and VLDL in insulin resistant cases as compared to sensitive subjects, with non-significant difference in terms of lipid profile measures such as total cholestrol, high density lipoprotein and low-density lipoprotein levels among them ( Table 4).

Insulin resistance is most prevalent in the second and third trimesters, when the foetal supply of nutrients is ensured. Insulin resistance causes dyslipidaemia in a variety of organs, including adipocytes and the liver, due to their high metabolic requirement. It inhibits lipoprotein lipase activity in adipocytes, resulting in an increase in free fatty acids and the release of inflammatory cytokines including IL-6 and TNF. Insulin resistance impairs glucose output, and fatty acid metabolism is changed, resulting in increased triglyceride accumulation and VLDL release from the liver. ^24^ This evidence corroborates our findings. Furthermore, compared to normal pregnant women, women with GDM had greater serum triacylglycerol concentrations but lower LDL-cholesterol concentrations. ^25^ Total cholesterol, HDL cholesterol, and apolipoprotein concentrations in GDM patients and control subjects were not substantially different in research by Nawal *et al.* ^26^ Hyperglycaemia and hyperlipidaemia stimulate the development of leptin, which decreases insulin production. ^27^ As a result, albeit being insignificant, there was a positive connection between leptin and all lipid markers in the current investigation. Interestingly, while low levels of circulating insulin are detected in insulin resistance due to dyslipidaemia, the present investigation found a strong negative connection between C peptide and total cholesterol and LDL. Leptin and TG have a positive association, but HDL and leptin have a negative correlation. ^28^ On comparing the lipid profile of insulin-resistant people with varied leptin and leptin receptor gene genotypes in our study, no significant difference was observed among them.

### Limitations of the study

The study's major flaw is its small sample size; however the study may be considered as a pilot study which can be extended to a larger population. Another limitation of the study was unequal number of insulin sensitive and resistant subjects. But, this was unavoidable as the definition of the groups was based on the calculation of insulin resistance, that is after enrolment of the subjects.

## Conclusion

As there are no previous studies done on the leptin/leptin gene receptor polymorphism in insulin resistance, this study elucidated the role of polymorphism of leptin gene in pregnant insulin resistant women. According to the findings of this study, there is no significant link between LEPG2548A and leptin receptor Gln223Arg alleles with insulin resistance in pregnancy. In comparison to other genotypes, persons with homozygous dominant, 'GG' of LEPR had increased insulin resistance and hyperinsulinemia. Those who have the homozygous dominant, 'AA' form of the leptin gene exhibited hyperglycaemia as compared to other genotypes. This study therefore concluded no established association of gene polymorphism in detecting insulin resistance in pregnancy. Hyperleptinemia in pregnancy also is not a contributory finding in predicting insulin resistance. Assaying C peptide, insulin, glucose and triglyceride levels in pregnant women is in routine practice. However, detecting gene polymorphisms of LEP and LEPR as markers to predict insulin sensitivity in advance, before starting insulin therapy, may be of therapeutic use, both from maternal as well as foetal point of view.

## Data availability

### Underlying data

Biostudies: Association of Leptin and Leptin receptor Gene Polymorphisms with Insulin Resistance in Pregnant Women: A Cross-Sectional Study. Accession number: S-BSST854. https://www.ebi.ac.uk/biostudies/studies/S-BSST854. ^29^

The project contains the following underlying data:

• [IR data.xlsx] (genotyping and metabolic parameters values).
