# Supplementary material for: Association of Leptin and Leptin receptor Gene polymorphisms with Insulin resistance in pregnant women: A cross-sectional study
Source: F1000Res. 2025 Mar 13;11:692. Originally published 2022 Jun 22. [Version 3] doi: 10.12688/f1000research.122537.3 (PMC9475205; doi:10.12688/f1000research.122537.3)
Supplement: Peer review history

# Reviewer report: Reviewer response for version 2

**DOI:** 10.5256/f1000research.138182.r141738

**Published:** 2025-03-10

**Authors:** Subramani Parasuraman

**Affiliations:** 1 Department of Pharmacology, Faculty of Pharmacy, AIMST University, Bedong, Malaysia

The purpose of the study must be justified in the introduction section. A total of 208 pregnant women were enrolled in the study. The method of sample size calculation must be described.

 The genetic analysis method should be described in detail. The results are well described. The study found no significant association between leptin and leptin receptor gene polymorphisms with insulin resistance in pregnancy, and the outcome can be justified through a strong literature review. Overall, it is a well-presented article and can be considered for acceptance after revision.

Is the work clearly and accurately presented and does it cite the current literature?

Yes

If applicable, is the statistical analysis and its interpretation appropriate?

Yes

Are all the source data underlying the results available to ensure full reproducibility?

Yes

Is the study design appropriate and is the work technically sound?

Yes

Are the conclusions drawn adequately supported by the results?

Partly

Are sufficient details of methods and analysis provided to allow replication by others?

Partly

Reviewer Expertise:

Pharmacology

I confirm that I have read this submission and believe that I have an appropriate level of expertise to confirm that it is of an acceptable scientific standard.

# Reviewer report: Reviewer response for version 2

**DOI:** 10.5256/f1000research.138182.r149777

**Published:** 2022-09-14

**Authors:** Rukmini Mysore Srikantiah

**Affiliations:** 1 Department of Biochemistry, Kasturba Medical College, Mangalore, Manipal Academy of Higher Education, Manipal, India

The revised version of this article is as per the requirements.

Is the work clearly and accurately presented and does it cite the current literature?

Partly

If applicable, is the statistical analysis and its interpretation appropriate?

Yes

Are all the source data underlying the results available to ensure full reproducibility?

Yes

Is the study design appropriate and is the work technically sound?

Yes

Are the conclusions drawn adequately supported by the results?

Partly

Are sufficient details of methods and analysis provided to allow replication by others?

Yes

Reviewer Expertise:

Dyslipidemia, Diabetes mellitus, Nutrition

I confirm that I have read this submission and believe that I have an appropriate level of expertise to confirm that it is of an acceptable scientific standard.

# Reviewer report: Reviewer response for version 2

**DOI:** 10.5256/f1000research.138182.r149776

**Published:** 2022-09-12

**Authors:** Suresh Kumar Srinivasamurthy

**Affiliations:** 1 Department of Pharmacology, Ras Al Khaimah College of Medical Sciences, Ras Al Khaimah Medical and Health Sciences University, Ras Al Khaimah, United Arab Emirates

The article is revised as required.

Is the work clearly and accurately presented and does it cite the current literature?

Partly

If applicable, is the statistical analysis and its interpretation appropriate?

Partly

Are all the source data underlying the results available to ensure full reproducibility?

Yes

Is the study design appropriate and is the work technically sound?

Yes

Are the conclusions drawn adequately supported by the results?

Partly

Are sufficient details of methods and analysis provided to allow replication by others?

Yes

Reviewer Expertise:

Medication errors, pharmacoepidemiology

I confirm that I have read this submission and believe that I have an appropriate level of expertise to confirm that it is of an acceptable scientific standard.

# Reviewer report: Reviewer response for version 1

**DOI:** 10.5256/f1000research.134537.r146106

**Published:** 2022-08-30

**Authors:** Rukmini Mysore Srikantiah

**Affiliations:** 1 Department of Biochemistry, Kasturba Medical College, Mangalore, Manipal Academy of Higher Education, Manipal, India

The authors have undertaken a significant molecular study which is helpful in assessing a possible link in leptin and leptin receptor gene polymorphisms and insulin resistance in pregnant women. The adopted methodology is relevant. The total number of pregnant women were 208, of which 74 were insulin resistant and 134 were insulin sensitive. The study would have been more impactful with the controls and the insulin resistant cases being almost equal. The authors have concluded that there is no significant association between leptin and leptin receptor gene polymorphisms with insulin resistance in pregnancy, and genotyping of these genes may be useful in predicting insulin resistance and gestational diabetes in pregnancy. 

 The comments by the approver 1 are relevant and must be incorporated.

 The manuscript also requires the following minor changes: 

1. The introduction and the methodology in the abstract needs mentioning the rationale for lipid profile parameters and correlation rather than in the results only. 
2. The insignificant finding of C- peptide, lipid profile, and leptin correlation with three alleles of leptin receptor gene (Table 6) can be explained with relevant references. For example, please see - Nielsen *et al.*, (2009) ^1^.

Is the work clearly and accurately presented and does it cite the current literature?

Partly

If applicable, is the statistical analysis and its interpretation appropriate?

Yes

Are all the source data underlying the results available to ensure full reproducibility?

Yes

Is the study design appropriate and is the work technically sound?

Yes

Are the conclusions drawn adequately supported by the results?

Partly

Are sufficient details of methods and analysis provided to allow replication by others?

Yes

Reviewer Expertise:

Dyslipidemia, Diabetes mellitus, Nutrition

I confirm that I have read this submission and believe that I have an appropriate level of expertise to confirm that it is of an acceptable scientific standard, however I have significant reservations, as outlined above.

## References

[1] : Pregnancy-induced rise in serum C-peptide concentrations in women with type 1 diabetes. *Diabetes Care* .2009;32(6) : 10.2337/dc08-1832 1052-7 10.2337/dc08-1832 19244092 PMC2681014

# Response

**Published:** 2022-09-01

**Authors:** Usha Adiga

**Affiliations:** Apollo institute of medical sciences & research Chittoor ,Andhra Pradesh, India, India

Comment 1: Modified as per reviewers suggestions.

 Comment 2: modified as per reviewers suggestion.

# Reviewer report: Reviewer response for version 1

**DOI:** 10.5256/f1000research.134537.r141739

**Published:** 2022-07-04

**Authors:** Suresh Kumar Srinivasamurthy

**Affiliations:** 1 Department of Pharmacology, Ras Al Khaimah College of Medical Sciences, Ras Al Khaimah Medical and Health Sciences University, Ras Al Khaimah, United Arab Emirates

1. The flow of the manuscript need to to be rechecked, as several lacunae exist especially while presenting genotype data of two genes (Table 6 gives data on only one gene). A table/data on genotypes of leptin gene and metabolic parameters similar to Table 6 is missing in manuscript. Also the language and grammar to be checked.

 2. Contradictory statements - Abstract mentions hyperglycaemia was observed in subjects with homozygous dominant, ‘AA’ of leptin gene (p=0.0173). However hyperglycemia is seen with heterozygous genotype of leptin receptor gene (Table 6).

 3. P value should be presented in uniform format; some places 'p=' where as in other 'p<'.

 4. Results section is been presented in methods section which needs correcting.

 5. Table 1 and Table 2 can be presented as texts (not tables) because it doesn't address the main objective.

 6. Footnote on tables to mention differences between one star and two stars (p values).

 7. Table 6 - p value for FBS is not marked with star.

 8. Table 6 - the data presented is of genotypes but the caption indicates alleles.

 9. Table 4 presenting demographics can be brought before Table 3.

 10. Check if there is any linkage disequilibrium between these two genes.

 11. Discussion needs to elaborate on the non-significance obtained in light of non-significant BMI among the groups.

Is the work clearly and accurately presented and does it cite the current literature?

Partly

If applicable, is the statistical analysis and its interpretation appropriate?

Partly

Are all the source data underlying the results available to ensure full reproducibility?

Yes

Is the study design appropriate and is the work technically sound?

Yes

Are the conclusions drawn adequately supported by the results?

Partly

Are sufficient details of methods and analysis provided to allow replication by others?

Yes

Reviewer Expertise:

Medication errors, pharmacoepidemiology

I confirm that I have read this submission and believe that I have an appropriate level of expertise to confirm that it is of an acceptable scientific standard, however I have significant reservations, as outlined above.

# Response

**Published:** 2022-07-05

**Authors:** Usha Adiga

**Affiliations:** Apollo institute of medical sciences & research Chittoor ,Andhra Pradesh, India, India

Table 6 represents the comparison of biochemical parameters of leptin gene and Table 7 represents that of leptin receptor gene which is missing. Table 7 is published as table 6. And table 6 is missing. This will be corrected.

 Once this correction is done, abstract also makes sense.

 All other corrections will be made as suggested.

 Linkage disequilibrium can be done between two SNPs of a single gene. But in this study, two separate genes were analyzed. Hence LD was not calculated.

# Response

**Published:** 2022-09-01

**Authors:** Usha Adiga

**Affiliations:** Apollo institute of medical sciences & research Chittoor ,Andhra Pradesh, India, India

Comment 1: modified and added the new table.

 Comment 2: modified, the contradiction statement is due to the missing table and the title of table is corrected.

 Comment 3: p value is presented in uniform format.

 Comment 4: modified as suggested.

 Comment 5: modified as suggested.

 Comment 6: footnote on *p value and **p value is stated under table.

 Comment 7: FBS value in the table 6 is added with *

 Comment 8: table 6 caption is modified.

 Comment 9: the data in the table is explicit hence it could not be represented as text.

 Comment 10: linkage disequilibrium cannot be done because its of two separate genes, single SNP has been done for each. 

 Comment 11: modified.
