# Supplementary material for: Association of Leptin and Leptin receptor Gene polymorphisms with Insulin resistance in pregnant women: A cross-sectional study
Source: F1000Res. 2022 Jun 22;11:692. [Version 1] doi: 10.12688/f1000research.122537.1 (PMC9475205; doi:10.12688/f1000research.122537.1)
Supplement: Peer review history

# Response

**Published:** 2022-09-01

**Authors:** Usha Adiga

**Affiliations:** KS HEGDE MEDICAL ACADEMY, India

Comment 1: Modified as per reviewers suggestions.

 Comment 2: modified as per reviewers suggestion.

# Response

**Published:** 2022-07-05

**Authors:** Usha Adiga

**Affiliations:** KS HEGDE MEDICAL ACADEMY, India

Table 6 represents the comparison of biochemical parameters of leptin gene and Table 7 represents that of leptin receptor gene which is missing. Table 7 is published as table 6. And table 6 is missing. This will be corrected.

 Once this correction is done, abstract also makes sense.

 All other corrections will be made as suggested.

 Linkage disequilibrium can be done between two SNPs of a single gene. But in this study, two separate genes were analyzed. Hence LD was not calculated.

# Response

**Published:** 2022-09-01

**Authors:** Usha Adiga

**Affiliations:** KS HEGDE MEDICAL ACADEMY, India

Comment 1: modified and added the new table.

 Comment 2: modified, the contradiction statement is due to the missing table and the title of table is corrected.

 Comment 3: p value is presented in uniform format.

 Comment 4: modified as suggested.

 Comment 5: modified as suggested.

 Comment 6: footnote on *p value and **p value is stated under table.

 Comment 7: FBS value in the table 6 is added with *

 Comment 8: table 6 caption is modified.

 Comment 9: the data in the table is explicit hence it could not be represented as text.

 Comment 10: linkage disequilibrium cannot be done because its of two separate genes, single SNP has been done for each. 

 Comment 11: modified.
